# Structural arrangement of the active back-to-back dimer in N-glycosylated ErbB receptors is regulated by heterodimerization

**DOI:** 10.22099/mbrc.2023.47147.1822

**Published:** 2023

**Authors:** Romina Mashayekh-Poul, Maryam Azimzadeh-Irani, Seyedeh Zeinab Masoomi-Nomandan

**Affiliations:** Faculty of Life Sciences and Biotechnology, Shahid Beheshti University, Tehran, Iran.

**Keywords:** ErbB receptors, Dimerization, Back-to-back dimer, Molecular modeling

## Abstract

The human epidermal growth factor receptor (EGFR/ErbB) family consists of four members (ErbB1-4) and belongs to the superfamily of receptor tyrosine kinases (RTKs). The ErbB family members participate in multiple cellular pathways and are the key players in several cancers (brain, breast, lung etc.). Activation of these family members depends on their extracellular domains forming back-to-back hetero/homo dimers. Moreover, dimers are glycosylated, which is a crucial post-translational modification that affects the conformation and function of the protein. Here, molecular modeling and molecular docking are used to comprehensively investigate the dimerization mechanism in glycosylated back-to-back active dimer formation in the entire ErbB receptors for the first time. Results showed that 21 out of 37 clusters of active back-to-back dimers formed by all family members are through heterodimerization. Including; ErbB1-ErbB3/ErbB4, ErbB2-ErbB3/ErbB4 and ErbB3-ErbB4. Ranking ErbB2-ErbB3 as the most stabilized back-to-back dimeric construct. While glycan arrangements favor both homo/hetero dimerization at the dimeric interfaces, it promotes heterodimerization by stabilizing and packing the ligand binding sites of EGFR and ErbB2 respectively. These findings pave the path to future heterodimeric interface/glycan targeting rational anti-cancer drug designs for ErbB receptors.

## INTRODUCTION

**Figure 1:**
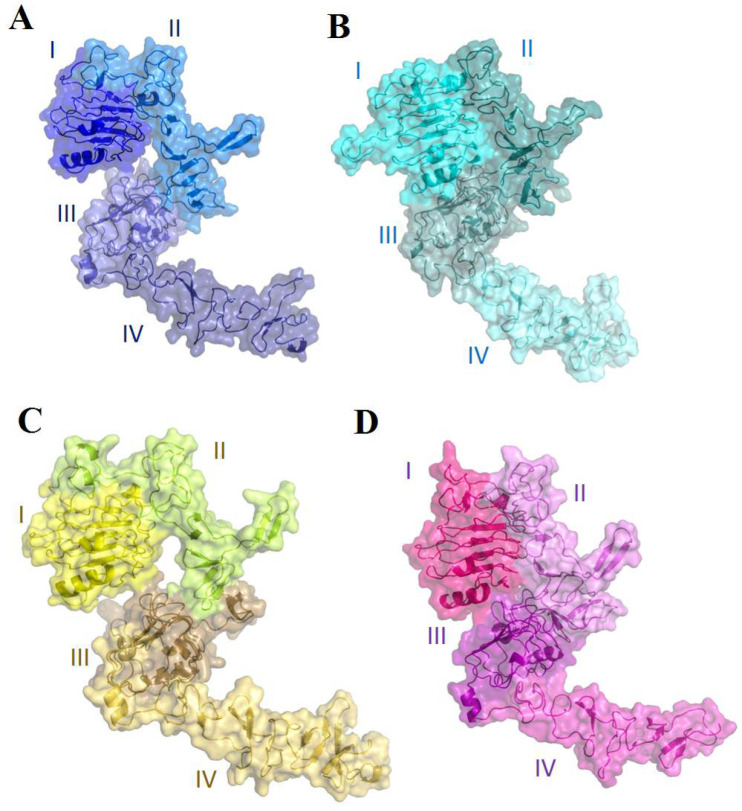
The extracellular domains of ErbB family members.

The human epidermal growth factor receptor (EGFR) family [1], also known as ErbBs or HERs [2], belongs to subclass 1 of the superfamily of receptor tyrosine kinase (RTKs) [3]. This family comprises four distinct receptors: EGFR/ErbB-1 [4, 5], HER2/ErbB-2 [6, 7], HER3/ErbB-3 [8, 9] and HER4/ErbB-4 [2, 10-11]. Based upon the cDNA analysis and the primary amino acid structure of EGFR [1 , 5], all members of the ErbB family consist of a large extracellular domain, a single-pass hydrophobic transmembrane α-helix, and an intracellular domain [3, 5, 12, 13]. The intracellular domain consists of a juxtamembrane part, a distinct tyrosine-protein kinase segment, and a tyrosine-rich carboxyterminal tail [14], and the extracellular domain contains four subdomains [3], including I/L1, II/CR1, III/L2 and IV/CR2 [6] (Fig. 1). Domains II and IV retain multiple cysteine residues that participate in disulfide bond formation and dimer formation with homologous receptors, and domains I and III are conveyed leucine-rich segments that partake in ligand binding [3, 14]. The extracellular domain is vital in dimerization due to a critical structural element of the dimerization interface called the dimerization arm in subdomain II [9]. Ligand binding to the extracellular region induces receptor dimerization and activation of the cytoplasmic kinase, leading to autophosphorylation and initiation of downstream signaling [6].

**A: **The extracellular domain of ErbB1. Subdomains I, II, III and IV are colored with blue, marine, slate and deep blue transparent surfaces and cartoons, respectively. **B:** The extracellular domain of ErbB2. Domains I, II, III and IV are colored with cyan, teal, light teal and aquamarine transparent surfaces and cartoons, respectively. **C:** The extracellular domain of ErbB3. Domains I, II, III and IV are colored with yellow, limon, sand and yellow-orange transparent surfaces and cartoons, respectively. **D: **The extracellular domain of ErbB4. Domains I, II, III and IV are colored with hot pink, violet, purple and light magenta transparent surfaces and cartoons, respectively.

The ErbB family members are expressed in many cells, including epithelial, mesenchymal, cardiac, and neuronal cells [13]. They critically participate in numerous cellular processes like proliferation, differentiation, survival, adhesion and migration [1, 11]. Overexpression and mutation of these proteins play a vital role in the development and progression of various diseases, such as breast or brain cancers, particularly in processes like epithelial-mesenchymal transition (EMT), migration, metastasis, drug resistance, and tumor invasion [1, 15]. Considering their essential role in tumor progression, they have been attractive targets for therapeutic interventions in the past few decades.

These receptors activation is entirely dependent on forming various dimers. During the activation mechanism, receptor dimerization is induced by binding ligands to receptors on the cell surface [11]. There are two distinct conformational states for ErbBs extracellular domain, extended active and tethered inactive forms. Without a ligand, an intramolecular inactive tether between domains II and IV is obscured. In the active conformation, the structure is extended and reveals a dimeric form and domains I and III are bridged by ligands [13]. The four family members can form two types of hetero and homodimers. Dimers that are formed between two identical or different family members are known as homodimers and heterodimers, respectively [11, 16]. They can form four homodimers and six heterodimers, including: ErbB1-ErbB1, ErbB2-ErbB2, ErbB3-ErbB3, ErbB4-ErbB4, ErbB1-ErbB2, ErbB1-ErbB3, ErbB1-ErbB4, ErbB2-ErbB3, ErbB2-ErbB4 and ErbB3-ErbB4.

In the active dimer, the overall structure of the ligand-bound extracellular domain is a stable back-to-back form [17]. This shape is formed from two kidney-shaped elements facing each other with their concave flanks [18]. Among the four family members, ErbB2 lacks ligand binding ability, and ErbB3 has a catalytically damaged kinase [19, 20]. However, if a specific receptor is overexpressed and in the presence of related ligands, its dimer formation can be biased. [15]. The interaction between ligands and receptors is essential in considerable aspects of morphogenesis [11]. The essentiality of dimer formation in the activation of the ErbB receptors and the complexity of homo/hetero dimerization among the family members has been a long-time challenge in the anti-tumor ErbB positive drug designs [14, 21]. 

The ErbB family is known to undergo various post-translational modifications (PTMs), including phosphorylation, glycosylation, acetylation, nitrosylation, methylation, and oxidation [38, 39]. However, only a few of these modifications are related to the extracellular domain, such as glycosylation and methylation [40-42]. Furthermore, glycosylation is the most crucial post-translational modification of the ErbB receptors that can increase the complexity and functioning of these proteins. Glycosylation involves the addition of carbohydrate moieties to specific sites of proteins and plays a crucial role in protein maturation and sorting [22-25]. Glycosylation of the extracellular domain affects the ErbBs activation, function and structural conformation[26]. Glycosylation of the ErbB receptors is critical for their stability, ligand binding, proper folding and dimerization [12, 26]. Also, it affects endocytosis and promotes heterodimer formation [12]. In recent years, the structural role of ErbBs glycosylation at the atomic level in dimer formation has attracted attention in the community [12, 23, 25-26]. N-glycosylation is the most critical type of glycosylation, which contains the attachment of carbohydrates to the Asparagines nitrogen atom, located within the Asn-X-Ser/Thr motif, where X is any amino acid but Proline [27]. N-glycosylation affects signaling, protein folding and flexibility [28]. Several N-glycosylation site Asparagines are located in the extracellular domain of different ErbB receptors within the consensus N-glycosylation sequence [26].

Understanding the two least studied yet most complex mechanisms of homo/heterodimer-ization and glycosylation could lead to developing novel anti-ErbB cancer therapeutics. In this study, molecular modeling and molecular dockings were performed to explore the mechanism of the receptors glycosylated extracellular domains dimerization. Results showed that the majority of the active back-to-back dimers formed by all ErbB family members are through heterodimerization. Including; ErbB1-ErbB3/ErbB4, ErbB2-ErbB3/ErbB4 and ErbB3-ErbB4. Ranking ErbB2-ErbB3 as the most stabilized back-to-back dimeric construct. While glycan arrangements favor both homo/hetero dimerization at the dimeric interfaces, it promotes heterodimerization by stabilizing and packing the ligand binding sites of EGFR and ErbB2 respectively.

## MATERIALS AND METHODS

**Construction of the Homo and heterodimers:** The X-ray crystal structures of the extracellular domain of the ErbB family members were obtained from the protein data bank [29]. PDB ID 3NJP for ErbB1, 1N8Y for ErbB2, 7MN5 for ErbB3 and 3U7U for ErbB4. Extended monomers without any connected ligand were extracted from these structures by using Pymol. Then, the missing amino acids of the PDB ID 7MN5 (263 to 276 and 323 to 326) were modeled with the SWISS-MODEL server [30] to complete the structure. Haddock 2.4 [31, 32] was used to dock the monomers of heterodimers and homodimers. In total, ten sets of molecular docking were performed to obtain all the plausible homo and heterodimers of the ErbB family including: ErbB1-ErbB1, ErbB2-ErbB2, ErbB3-ErbB3, ErbB4-ErbB4, ErbB1-ErbB2, ErbB1-ErbB3, ErbB1-ErbB4, ErbB2-ErbB3, ErbB2-ErbB4 and ErbB3-ErbB4 dimers. HADDOCK output contains many parameters like HADDOCK score, RMSD, Z-Score, cluster size, Van der Waals energy, Electrostatic energy, etc. These parameters are utilized for analyzing the structures. ErbB1-ErbB1, ErbB2-ErbB2, ErbB3-ErbB3, ErbB4-ErbB4, ErbB1-ErbB2, ErbB1-ErbB3, ErbB1-ErbB4, ErbB2-ErbB4 and ErbB3-ErbB4 had 2,1,6,7,0,3,4,5 and 7 back-to-back form clusters, respectively. HADDOCK score was used to select the best format for dimers with more than one back-to-back form. Chosen back-to-back forms have cluster sizes ranging from 4 to 18 and Z-Scores ranging from -1.5 to -0.1. PyMOL was used for biomolecular visualization of the structures. 

**Glycosylation of the dimeric constructs:** Glycosylation of the ErbB family is a complex and diverse process that is influenced by various physiological and pathological conditions [26,27,34]. In order to investigate the glycosylation of the ErbB family, we used a core model that is observed in multiple human proteins, and many studies have investigated it [34]. EGFR, ErbB2, ErbB3 and ErbB4 monomers were N-glycosylated by the GLYCAM builder [33]. The model was glycosylated using high mannose oligosaccharides (DManpa1-3[DManpa1-6]DManpb1-4DGlcpNAcb1-4DGlcpNAcb1-OH) and glycans, which end with serine or threonine (N-X-S/T where X is any amino acid except Proline [27]). ErbB1, ErbB2, ErbB3 and ErbB4 have 10, 8, 10 and 8 glycosylation sites respectively (Table 1). Glycosylated heterodimers and homodimers were then docked manually using Pymol's align tool. The HADDOCK outputs were used as the template for the superpositioning of the glycosylated monomers to build the glycosylated dimers. Glycosylation was examined at two sites; The first site is the dimeric interface, and the second site is the ligand binding site (Fig. S1). The dimeric interface where two monomers interact is between domains II and IV. Also, ligand binding site is located chiefly between domains I and III. Then, the dimeric structures were investigated by comparing the number of their attached glycans.

**Table 1:** List of glycosylated Asparagines in the ErbB family monomers

**Monomer**	**Location**
**ErbB1**	ASN104, ASN151, ASN172, ASN337, ASN389, ASN420, ASN504, ASN544, ASN579, ASN599
**ErbB2**	ASN45, ASN101, ASN102, ASN164, ASN236, ASN507, ASN548, ASN606
**ErbB3**	ASN99, ASN223, ASN326, ASN381, ASN387, ASN410, ASN442, ASN495, ASN539, ASN589
**ErbB4**	ASN149, ASN228, ASN333, ASN385, ASN448, ASN470, ASN523, ASN551

## RESULTS

**Figure 2:**
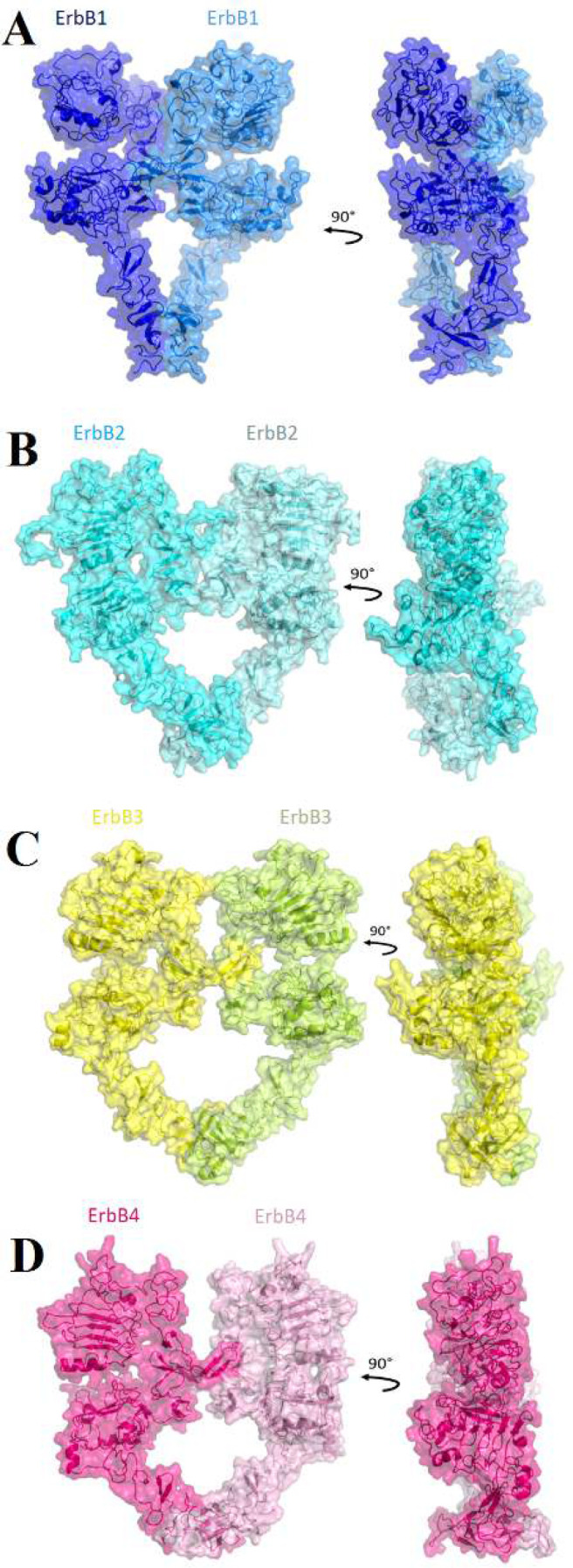
Structural presentation of back-to-back homodimers**. **The first and second monomers of ErbB1, ErbB2, ErbB3, and ErbB4 are shown with blue, marine, cyan, aquamarine, yellow, limon, hot pink, and pink transparent surfaces and cartoons, respectively.** A: **ErbB1-ErbB1 homodimer.** B: **ErbB2-ErbB2 homodimer.** C: **ErbB3-ErbB3 homodimer.** D: **ErbB4-ErbB4 homodimer.

**Figure 3:**
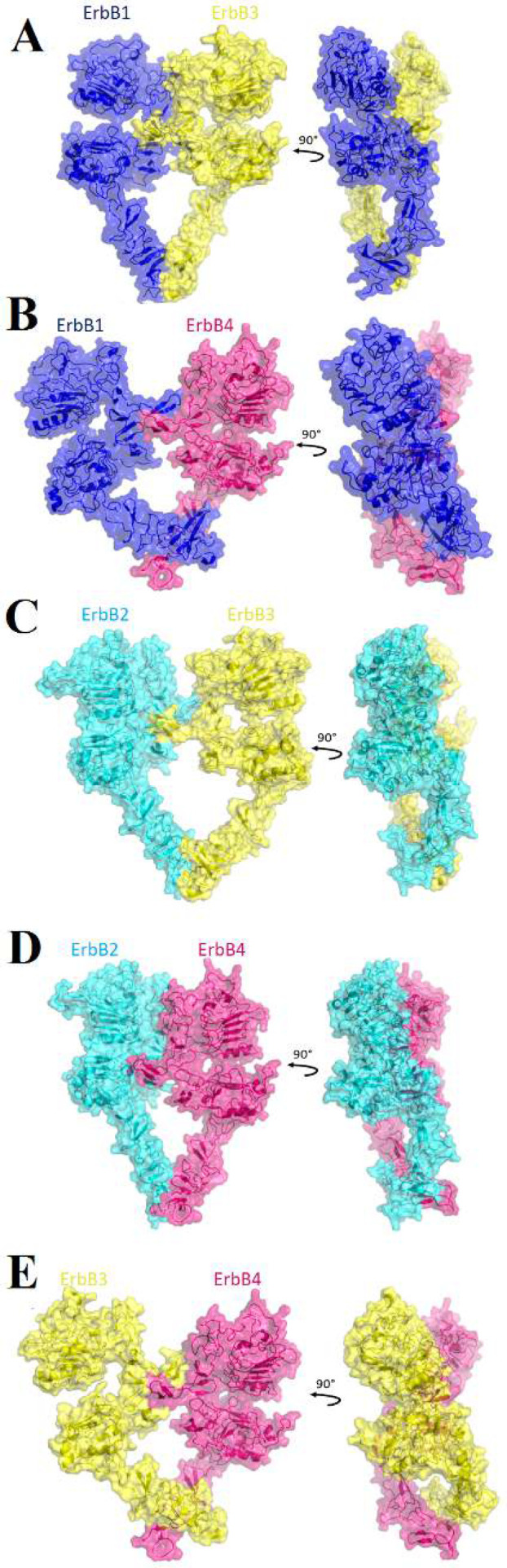
Structural presentation of back-to-back heterodimers. ErbB1, ErbB2, ErbB3, and ErbB4 are shown with blue, cyan, yellow and hot pink transparent surfaces and cartoons, respectively. Pictures A to E consecutively shown ErbB1-ErbB3, ErbB1-ErbB4, ErbB2-ErbB3, ErbB2-ErbB4 and ErbB3-ErbB4 heterodimers.

A total of 37 back-to-back dimers were constructed by molecular docking. Of which 16 were homodimers and 21 were heterodimers, with HADDOCK scores ranging from -16 to 178. Four homodimers and five heterodimers with the lowest HADDOCK score were selected for further investigations (Fig. 2 and 3). ErbB3-ErbB4 heterodimer with eight back-to-back clusters owns the most back-to-back active structures. ErbB2-ErbB2 homodimer and ErbB2-ErbB3 with one back-to-back cluster for each have the least active structures. ErbB1, ErbB3, and ErbB4 homodimers had two, six and seven back-to-back clusters. Also, ErbB1-ErbB3, ErbB1-ErbB4, and ErbB2-ErbB4 heterodimers had three, four and five back-to-back clusters, respectively. 

ErbB1-ErbB2 did not have any back-to-back dimers (Fig. S2). In our findings, we observed that ErbB1 and ErbB2 didn't exhibit the formation of back-to-back dimers. This suggests that the interaction between these two proteins with these attached sites occurs through alternative configurations such as back-to-head forms (Fig. 3). These are important findings to implicate for understanding the signaling and function of these receptors. One sample of a back-to-head form for each dimer with the lowest HADDOCK score (Table S1) is illustrated in Figure S2. Considering the HADDOCK score, on average, homodimers present a lower score than heterodimers (Table 2). In order to find the best structure, HADDOCK score, RMSD value, Van der Waals energy and Electrostatic energy were considered. The HADDOCK score ranged from -15.6 to 177.6, and the RMSD value went from 1.3 to 42.1 Å, while the Van der Waals energy varied from -99.8 to -56.7 kcal/mol, and Electrostatic energy ranged from -544.4 to -193.9 (Table 2). If the HADDOCK score is considered, the ErbB1-ErbB1 homodimer with a score of -16 is the best structure. While considering the RMSD values, the ErbB2-ErbB3 heterodimer with 1.3 Å has the highest docking stability. Evaluating the electrostatic energy, the ErbB1-ErbB1 homodimer with the lowest energy of -544.4 kcal/mol is the best structure. Still, if Van der Waals energy is considered, the ErbB1-ErbB4 heterodimer has the lowest energy of -99.8 kcal/mol (Table 2). The RMSD value of ErbB1-ErbB1 and ErbB1-ErbB4 dimer is 9.4 and 37.9 Å. Also, the ErbB2-ErbB3 heterodimer has Electrostatic energy of -193.9 kcal/mol and Van der Waals energy of -58.2 kcal/mol. While the ErbB1-ErbB1 homodimer has Van der Waals energy of -56.7 kcal/mol, and the ErbB1-ErbB4 heterodimer has Electrostatic energy of -285.6 kcal/mol. By putting these together, we can conclude that the ErbB1-ErbB1, ErbB1-ErbB4 and ErbB2-ErbB3 dimers are considered the best structures among all dimers Because their structures have the lowest energies and RMSD values.

**Table 2:** HADDOCK dimerization results in back-to-back forms

**PDB ID**	**Name**	**Cluster No.**	**HADDOCK score**	**Electrostatic energy (kcal/mol)**	**Van der Waals energy (kcal/mol)**	**RMSD (Å)**	**Cluster size**	**Z-Score**
3NJP-3NJP	ErbB1-ErbB1 homodimer	11	-15.6	-544.4	-56.7	9.4	4	-0.7
1N8Y-1N8Y	ErbB2-ErbB2 homodimer	13	125.1	-538.4	-60.2	38.8	4	-1.0
7MN5-7MN5	ErbB3-ErbB3 homodimer	13	114.3	-223.0	-94.6	42.1	4	-1.0
3U7U-3U7U	ErbB4-ErbB4 homodimer	2	41.3	-431.7	-96.6	39.6	18	-0.5
3NJP-7MN5	ErbB1-ErbB3 heterodimer	3	53.6	-466.4	-79.0	18.7	10	-1.2
3NJP-3U7U	ErbB1-ErbB4 heterodimer	2	66.6	-285.6	-99.8	37.9	15	-0.1
1N8Y-7MN5	ErbB2-ErbB3 heterodimer	3	177.6	-193.9	-58.2	1.3	6	-0.5
1N8Y-3U7U	ErbB2-ErbB4 heterodimer	10	116.6	-463.6	-78.2	1.9	4	-1.5
7MN5-3U7U	ErbB3-ErbB4 heterodimer	1	157.9	-231.7	-84.1	12.0	15	-0.4

**Figure 4:**
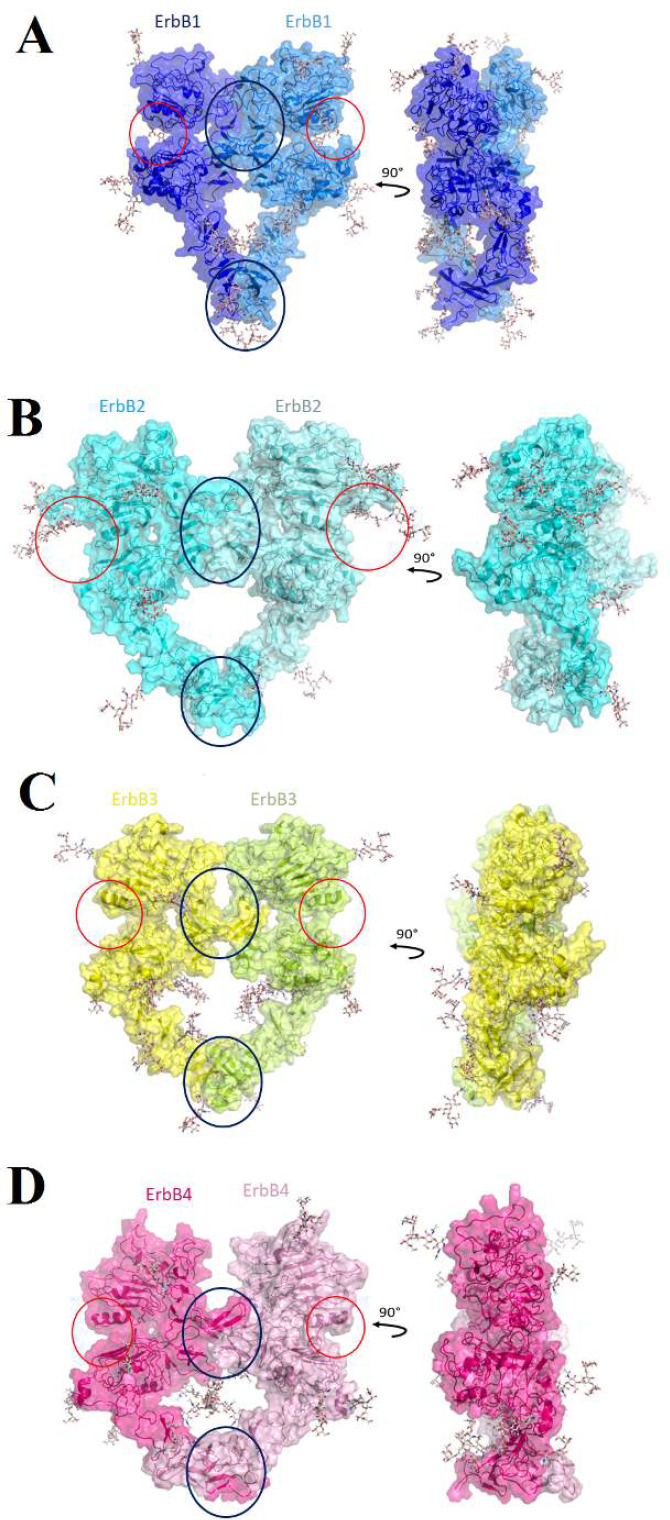
Glycosylated homodimers.

**Figure 5:**
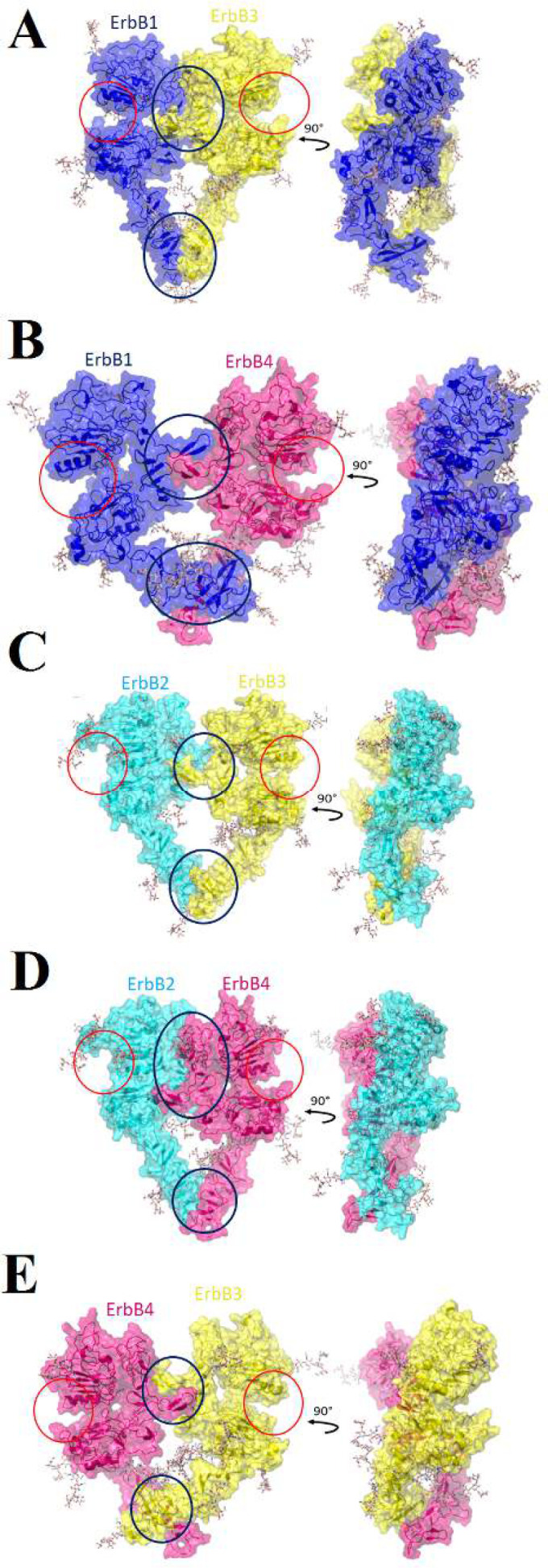
Glycosylated heterodimers.

The ErbB1, ErbB2, ErbB3, and ErbB4 have 10, 8, 10 and 8 attached glycans, respectively (Table 1). ErbB1-ErbB1, ErbB2-ErbB2, ErbB3-ErbB3, ErbB4-ErbB4, ErbB1-ErbB3, ErbB1-ErbB4, ErbB2-ErbB3, ErbB2-ErbB4 and ErbB3-ErbB4 dimers have 20, 16, 20, 16, 20, 18, 18, 16 and 20 glycans, respectively (Fig. 4 and 5). Four glycans are attached to the dimeric interface for all nine dimers except the ErbB1-ErbB4 heterodimer. ErbB1-ErbB4 heterodimer has three attaches glycans in the dimeric interface. The ErbB2-ErbB2 homodimer with six glycans has the most attached glycan in the ligand binding site. As mentioned above, the ErbB1-ErbB4 heterodimer with three glycans has the least attached glycan in the dimeric interface (Table 3). Glycans stabilize structures by forming hydrogen bonds [12, 17]. Overall, as the number of glycans increases, the connections become stronger, and as they decrease, the connections become weaker. According to this point, in the ligand binding site, the ErbB2-ErbB2 dimer has the strongest connections, while the ErbB1-ErbB4 dimer has the weakest connections in the dimeric interface.

The first and second monomers of ErbB1, ErbB2, ErbB3, and ErbB4 are colored with blue, marine, cyan, aquamarine, yellow, limon, hot pink, and pink transparent surfaces and cartoons, respectively. Also, blue and red circles are shown dimeric interfaces and ligand binding sites, respectively.

**Table 3:** Glycosylation results. The number of attached glycans in the dimeric interface and ligand binding site, along with the residue number of each monomer's glycans, are shown below.

**Name**	**Dimeric interface (D)**	**Ligand binding site (L)**	**Residue number**	
			**Monomer1**	** Monomer2**
**ErbB1-ErbB1 homodimer**	4	2	**D:** ASN579 – ASN599 **L:** ASN151	**D:** ASN579 – ASN599 **L:** ASN151
**ErbB2-ErbB2 homodimer**	4	6	**D:** ASN236 – ASN606 **L: **ASN45 – ASN101 – ASN102	**D:** ASN236 – ASN606 **L: **ASN45 – ASN101 – ASN102
**ErbB3-ErbB3 homodimer**	4	0	**D:** ASN223 – ASN589 **L: **-	**D:** ASN223 – ASN589 **L: **-
**ErbB4-ErbB4 homodimer**	4	0	**D:** ASN228 – ASN551 **L: **-	**D:** ASN228 – ASN551 **L: **-
**ErbB1-ErbB3 heterodimer**	4	1	**D:** ASN579 – ASN599 **L: **ASN151	**D:** ASN223 – ASN589 **L: **-
**ErbB1-ErbB4 heterodimer**	3	1	**D:** ASN579 **L: **ASN151	**D:** ASN523 – ASN551 **L: **-
**ErbB2-ErbB3 heterodimer**	4	3	**D:** ASN236 – ASN606 **L: **ASN45 – ASN101 – ASN102	**D:** ASN223 – ASN589 **L: **-
**ErbB2-ErbB4 heterodimer**	4	3	**D:** ASN236 – ASN606 **L: **ASN45 – ASN101 – ASN102	**D:** ASN228 – ASN551 **L: **-
**ErbB3-ErbB4 heterodimer**	4	0	**D:** ASN589 **L: **-	**D:** ASN149 – ASN523 – ASN551 **L: **-

## DISCUSSION

Four members of the ErbB family, including ErbB1, ErbB2, ErbB3 and ErbB4, consist of an extracellular domain [1], which binds to ligands and leads to the activation of the receptors [6]. The members of the ErbB family exhibit a sequence similarity of approximately 40-45% [45, 46]. Also, it is considerable that the ErbB family of proteins are large receptors consisting of three distinct sections: the extracellular domain that was mentioned earlier, transmembrane portion, and intracellular domain with a total number of 614, 619, 630, and 597 Amino acids in ErbB1-4 monomers extracellular domains, respectively [2, 4, 6, 9]. While this protein family is subject to multiple modifications and mutations, particularly in members with antibodies like ErbB1 [43], research has demonstrated that the overall 3D structure of these proteins remains largely unchanged. In fact, studies have indicated that the dimeric interfaces and intracellular domains of ErbB proteins are conserved and do not exhibit significant mutations [44]. And as our research is focused on the dimeric interfaces, the analyses presented here are  reliable.

These members are expressed in various cells and play an essential role in cellular processes [1, 11, 13]. Activation of receptors induced by binding the ligands to the ligand binding site in domains I and III and depends on dimer forming [12-14]. These four members can have homodimerization and heterodimerization based on the dimerization formed between identical or different family members. The overall stable structure in an active dimer is a back-to-back or heart-shaped form [17]. 

This study aimed to find the active back-to-back dimers and explore the glycosylation on the extracellular domains of the ErbB family by performing molecular dockings. The docking dimerization results showed that heterodimerization is more frequent in the ErbB family compared to homodimerization. Numerous previous studies suggested that heterodimerization could be more critical and could stabilize cell signaling that leads to cancers and tumors [12, 34, 35]. However, a comprehensive mechanism for the entire family members was not established, and studies mostly worked on one or two dimers. But here, all ten dimers are incorporated for the first time. Homodimerization of the ErbB1 is considered an important type of dimerization due to the fact that ErbB1 is the first known member of the family [1, 36]. Many experimental and computational studies modelled the ErbB1-ErbB1 dimer; in this study, our dimer has the best HADDOCK score and electrostatic energy (Table 2). Also, ErbB1 is one of the family members whose co-overexpression has a significant known role in cancer prognosis [34]. Considering that, using the data of its activated dimer could help scientists to find a valuable and practical anti-ErbB1 targeted therapy. ErbB2 and ErbB3 have preferred heterodimerization partners because ErbB2 lacks a ligand, and ErbB3 has a damaged kinase domain [19, 20, 36] and considering the docking dimerization results, the ErbB2-ErbB3 heterodimer has the best RMSD value (Table 2). Moreover, this heterodimer has eight back-to-back dimers, which is the most significant number in our results. 

ErbB1, ErbB2, ErbB3, and ErbB4 are illustrated with consecutive blue, cyan, yellow and hot pink transparent surfaces and cartoons. Blue and red circles are shown dimeric interfaces and ligand binding sites, respectively.

It is noteworthy that the X-ray crystal structure of some of the dimers is already available [2, 4, 6, 9]. Considering the fact that ErbB1 and ErbB3 are taken from a dimeric construct, it is more likely that they form the more favorable dimers in the docking results. Yet, we exclusively employed the extended monomers for all receptor structures, not their sites, interfaces, etc. Assuming these prints, we are confident that a reasonable chance was given to each receptor and our results are trustable. 

N-glycosylation, the most important glycosylation type, contains carbohydrates' attachment to the Asparagines nitrogen atom, located within the Asn-X-Ser/Thr motif [12, 23, 25-27]. Glycosylation (specially N-glycosylation) of the extracellular domain of the ErbB family affects their stability, activation, function, ligand binding and dimerization [28]. Computational studies mentioned that glycosylation increases the stability of this structure [12, 23, 34] and leads to stronger connections. Considering this point, fewer glycans means weaker connections. In this study, the ErbB1-ErbB4 heterodimer has the least attached glycans (three) in the dimeric interface, which means it has the weakest links in the dimeric interface among the nine glycosylated dimers. Due to the best Van der Waals energy, the ErbB1-ErbB4 heterodimer is one of the most potent ErbB heterodimers [34]. Also, ErbB1 and ErbB4 are considered fully functional family members [36].

Regarding the influence of glycans on connection strength, the six attached glycans in the ligand-binding site of ErbB2-ErbB2 homodimer cause strong connections, which can justify that ErbB2 lack of ability for ligand binding [12]. Many studies declared that the ErbB family members' misregulation significantly affects cancer development and progression [1, 15] and they are vital targets for cancer treatments [37]. Here, all members of the family are investigated for the first time, and results show that heterodimerization is more frequent and glycosylation promotes it. Considering the importance of these receptors in cancers, these results are useful to find and design new treatments against these conditions.

## Conflict of Interest:

The authors declare no competing interests.

## Supplementary materials
