# Supplementary material for: Structural arrangement of the active back-to-back dimer in N-glycosylated ErbB receptors is regulated by heterodimerization
Source: Mol Biol Res Commun. 2023;12(3):95–107. doi: 10.22099/mbrc.2023.47147.1822 (PMC10387173; doi:10.22099/mbrc.2023.47147.1822)
Supplement: Supplementary file 1 [file mbrc-12-095-s001.pdf]

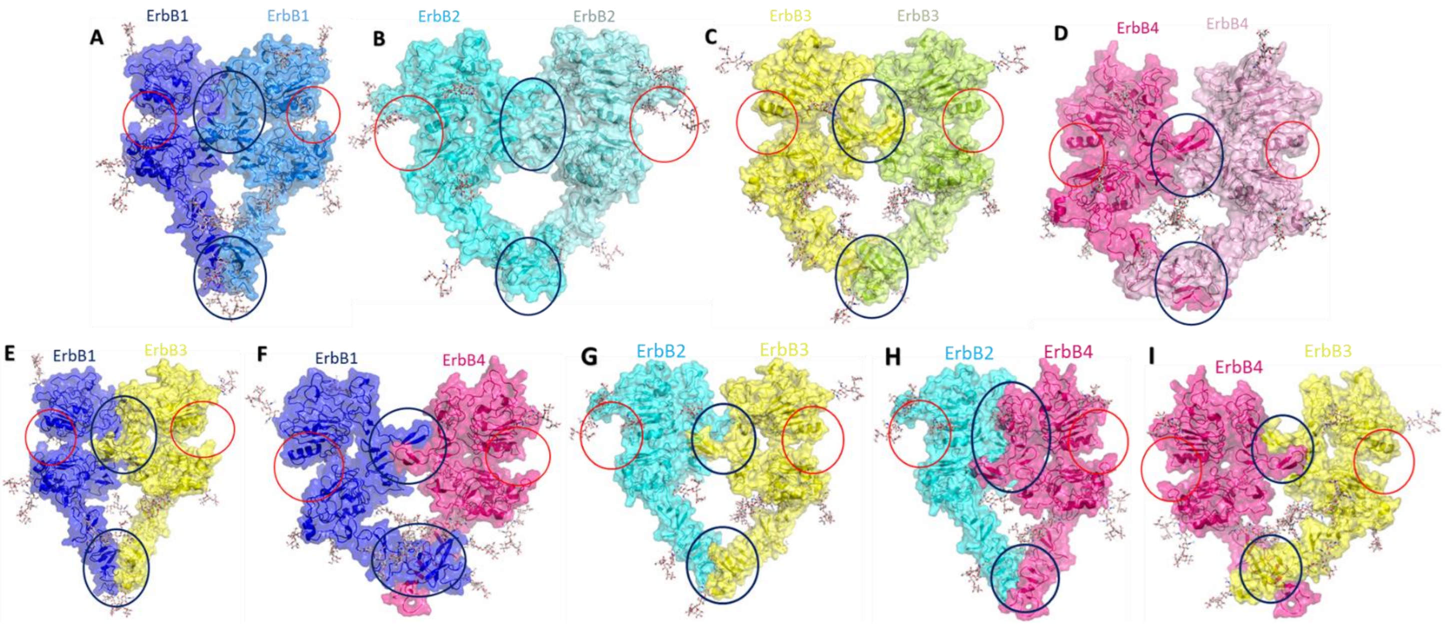

Figure S1. Dimeric interfaces and ligand binging sites in glycosylated dimers.

In A, B, C and D, the first and second monomers of ErbB1, ErbB2, ErbB3, and ErbB4 are colored with blue, marine, cyan, aquamarine, yellow, limon, hot pink, and pink transparent surfaces and cartoons, respectively. In E, F, G, H and I, ErbB1, ErbB2, ErbB3, and ErbB4 are illustrated with consecutive blue, cyan, yellow and hot pink transparent surfaces and cartoons. Moreover, blue and red circles are shown dimeric interfaces and ligand binding sites in all dimers, respectively.

Table S1. HADDOCK score and cluster number of back-to-head forms.

Name	Cluster No.	HADDOCK score
**ErbB1-ErbB1 homodimer**	1	-65.8
**ErbB2-ErbB2 homodimer**	8	96.0
**ErbB3-ErbB3 homodimer**	3	54.2
**ErbB4-ErbB4 homodimer**	7	-113.9
**ErbB1-ErbB2 heterodimer**	2	76.2
**ErbB1-ErbB3 heterodimer**	4	35.2
**ErbB1-ErbB4 heterodimer**	5	-17.2
**ErbB2-ErbB3 heterodimer**	1	131.3
**ErbB2-ErbB4 heterodimer**	5	125.7
**ErbB3-ErbB4 heterodimer**	7	125.5

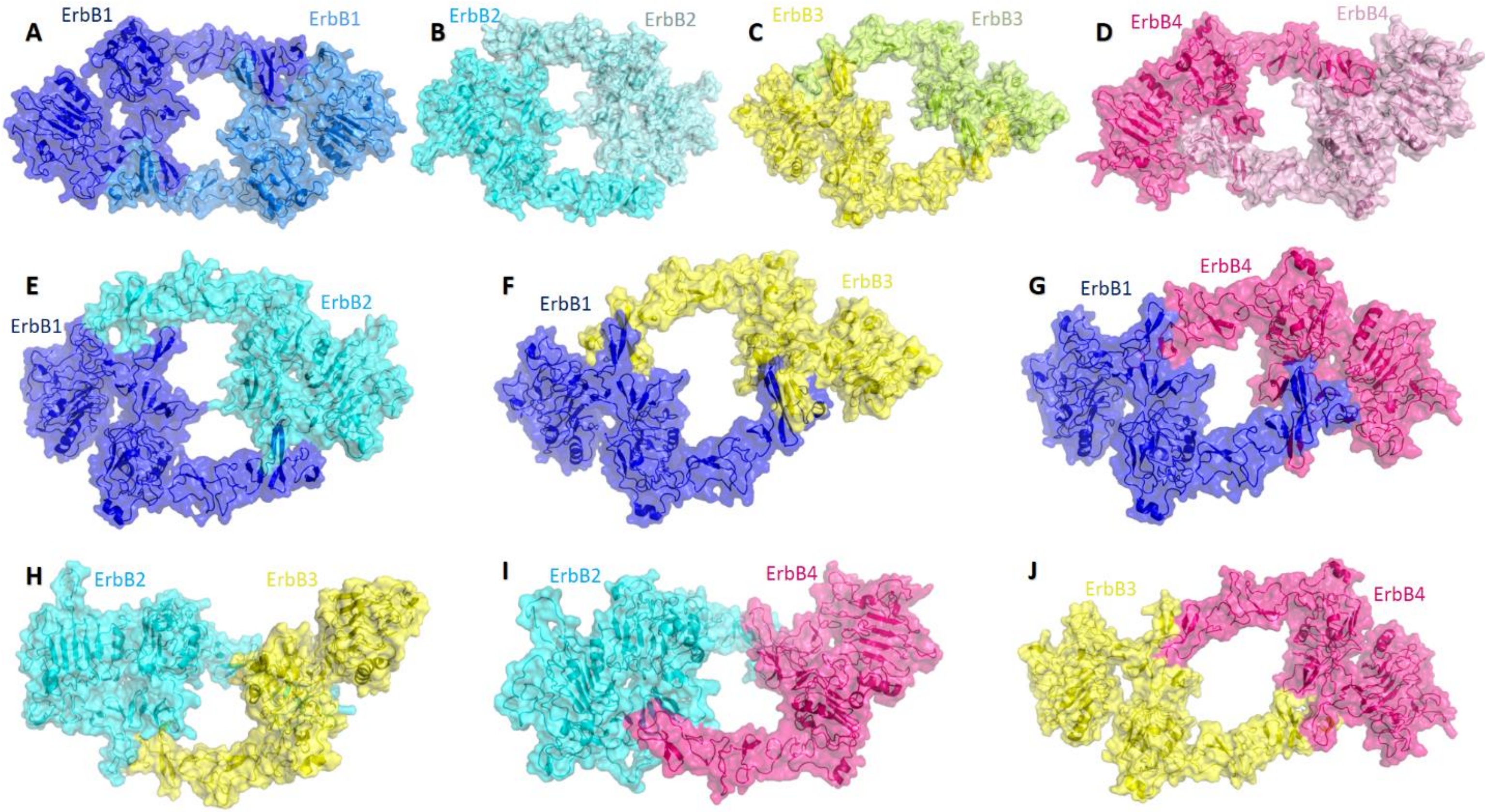

Figure S2: Structural presentation of back-to-head dimers.

For A, B, C and D, the first and second monomers of ErbB1, ErbB2, ErbB3, and ErbB4 are shown with blue, marine, cyan, aquamarine, yellow, limon, hot pink, and pink transparent surfaces and cartoons, respectively. Also, for E, F, G, H, I and J, ErbB1, ErbB2, ErbB3, and ErbB4 are colored with blue, cyan, yellow and hot pink transparent surfaces and cartoons, respectively. A: ErbB1-ErbB1 homodimer, cluster 1. B: ErbB2-ErbB2 homodimer, cluster 8. C: ErbB3-ErbB3 homodimer, cluster3. D: ErbB4-ErbB4 homodimer, cluster 7. E: ErbB1-ErbB2 heterodimer, cluster 2. F: ErbB1-ErbB3 heterodimer, cluster 4. G: ErbB1-ErbB4 heterodimer, cluster 5. H: ErbB2-ErbB3 heterodimer, cluster 1. I: ErbB2-ErbB4 heterodimer, cluster5. J: ErbB3-ErbB4 heterodimer, cluster 7.
